# Additional evidence that contour attributes are not essential cues for object recognition

**DOI:** 10.1186/1744-9081-4-26

**Published:** 2008-07-01

**Authors:** Ernest Greene

**Affiliations:** 1 Laboratory for Neurometric Research, Department of Psychology, University of Southern California, USA; 2 Neuropsychology Foundation, Los Angeles, CA, USA

## Abstract

It is believed that certain contour attributes, specifically orientation, curvature and linear extent, provide essential cues for object (shape) recognition. The present experiment examined this hypothesis by comparing stimulus conditions that differentially provided such cues. A spaced array of dots was used to mark the outside boundary of namable objects, and subsets were chosen that contained either contiguous strings of dots or randomly positioned dots. These subsets were briefly and successively displayed using an MTDC information persistence paradigm. Across the major range of temporal separation of the subsets, it was found that contiguity of boundary dots did not provide more effective shape recognition cues. This is at odds with the concept that encoding and recognition of shapes is predicated on the encoding of contour attributes such as orientation, curvature and linear extent.

## Background

"Stationary visual percepts, a tree, a stone, or a book, are as a rule extremely reticent as to the nature of the neural events which underlie their existence. We may hope to learn more about brain correlates if we turn to instances in which percept processes seem to be in a more active state." Kohler [1]

Cognitive, computational and neural theories of object recognition all share the concept that essential cues are provided by the orientation, curvature and linear extent of the lines and edges that lie at the boundary of an object and its component parts. We can describe these cues as "contour attributes," and describe the mechanisms for registering and encoding these attributes as "contour filters."

A previous study from this laboratory [2] offered evidence and arguments against the proposition that contour attributes provide the essential cues for object recognition. That study displayed dots that were positioned on the outer boundary of namable objects, varied the number of dots that were displayed in progressively larger samples, and manipulating the spatial positioning of dots within those samples. The display dots were chosen to provide subsets that either: a) formed contiguous strings that approximated line segments, b) were at randomly selected positions around the boundary of the shape, or c) were at evenly spaced positions around the boundary. For each condition the number of dots to be displayed (as a percentage of the total number of dots in the perimeter) was increased until the participant identified the object. The greatest percentage of dots was required for recognition when the subsets formed contiguous strings, and the smallest percentage was needed when the dots in the subsets were positioned with even spacing. The contiguous strings would have delivered the most information about contour attributes, yet this treatment condition provided the least effective cues for eliciting recognition of the shapes.

There were two additional reasons that the previous results [2] were at odds with the concept that contour attributes provide essential cues for recognition. First, it is widely believed that orientation-selective cells in primary visual cortex serve as the basic filters that register the contour attributes [3,4]. Yet recognition of many shapes was possible with only a small sampling of dots, and with the space between adjacent dots being wider than the receptive fields of orientation-selective cells. Second, even if one proposed new principles for registering alignments among these dots, it is not obvious how one would know which dots to connect.

The present experiments used the minimal transient discrete cue (MTDC) protocol [5-7] to examine whether dot subsets that should activate contour filters (because of spatial contiguity) provide better shape cues than do noncontiguous subsets. This protocol uses brief and successive display of subsets that were chosen from the full inventory of boundary dots. Successful recognition of the shape required integration of the information provided by each subset, and the level of performance reflected the degree to which that information was useful. The prior research [6,7] found that millisecond-level separation of subsets produced significant declines in recognition. This was the case even when the total display time for a given shape – and thus duration of cue persistence – was controlled. This means that the effectiveness of the shape cues, and their ability to be integrated, are reflected in the rate at which recognition declines when time differentials are inserted between the successive cues.

For the present experiment, the subsets provided either contiguous sequences of four dots, or four dots chosen at random locations in the boundary. Each contiguous dot subset would provide information about the local contour attributes of the shape. Each random dot subset would not provide this information, and to the extent that the subset might activate contour filters, would deliver inappropriate cues regarding alignments of the boundary. Therefore, to the extent that contour attributes are essential cues for shape recognition, performance levels should be higher for contiguous than for random subsets. The experimental results did not support this prediction.

## Methods

The apparatus for display of shapes (display board) was the same as used in earlier experiments [2,5,6]. Briefly outlined here, it consisted of a 64 × 64 array of red LEDs. The participant sat at a distance of 3.5 m from the display board, and at this distance, the diameter of each element and the center-to-center spacings were 4.95 and 7.42 arc', respectively. The horizontal and vertical dimensions of the full array were 7.74 × 7.74 arc°.

The circuits of the display board provided for activation of a given LED by specifying an x, y address position within the array, under the control of a microprocessor running with a clock speed of 24 Mhz. Rise and fall time for emission was in the range of 100 ns. Room illumination was from standard ceiling-mounted fluorescent fixtures that were fitted with opaque panels to block most of the light. This provided ambient illumination of 13.3 lux. Luminance of an emitting LED was set at 7 Cd/m^2^.

**Figure 1:**
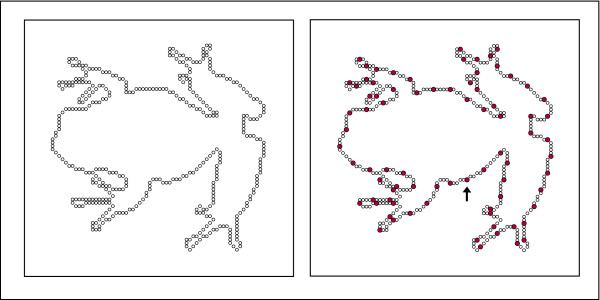
The left panel uses unfilled dots to show the positions of the full inventory of dots that marked the boundary for one of the shapes. This full inventory was never displayed. Rather, a sampling of dots, designated as the display set, was shown, using a sample size that was expected to yield recognition on approximately 75% of the trials. An example of a display set is shown by the filled dots in the right panel. For a given display, a random starting point was picked from among the list of addresses, shown by the arrow, and then every Nth dot location was selected for inclusion in the display set (with the value of N being that which would yield the number of dots needed in the display set). The size of display dots has been exaggerated for purposes of illustration.

The experiment displayed 64 shapes, these being the same as listed in Table 1 of reference [2], but with the elimination of five shapes in order to provide an equal number of shapes for each treatment condition. Each shape was represented by discrete boundary dots that formed a connected string of adjacent positions within the 64 × 64 array. Boundary dots for one shape, the frog, are illustrated in the left panel of Fig. 1.

As implemented here, the MTDC protocol was as follows. For a given shape, only some dots from the full inventory were shown, these being designated as the display set. The size of the display set was determined on the basis of testing done with other subjects. These tests established the number of evenly spaced dots needed to provide a 75% hit rate, *i.e.*, successful recognition of a given shape by 75% of the participants, if all the dots were simultaneously displayed. This might be regarded as providing a given subject with a 75% probability of identifying the shape, and if only for convenience, it may be described in this manner in what follows.

To specify the display set for each shape (done independently for each participant), the first address to be included in the set was chosen at random. From there, counting in a clockwise direction, every N^th ^address was chosen, the value of N being that which would yield the 75% hit rate. An example of one possible display set is illustrated in the right panel of Fig. 1.

Each display set was further broken into randomly chosen subsets containing four dots each (with one residual subset potentially having fewer than four). Spatial positioning of dots within the subsets provided the experimental treatment designated as "proximity," and temporal separation of subsets provided the treatment designated as "temporal separation," also known as T3.

There were two levels of the proximity condition, requiring either that the dots of the subset be contiguous, or that they be randomly selected from among the members of the display set. Note that contiguity is relative, in that the display set consists of every N^th ^position within the full inventory of boundary dot positions. For the random condition, there was an additional restriction that each dot in the subset must lie at least three steps away from other positions within the display set.

**Figure 2:**
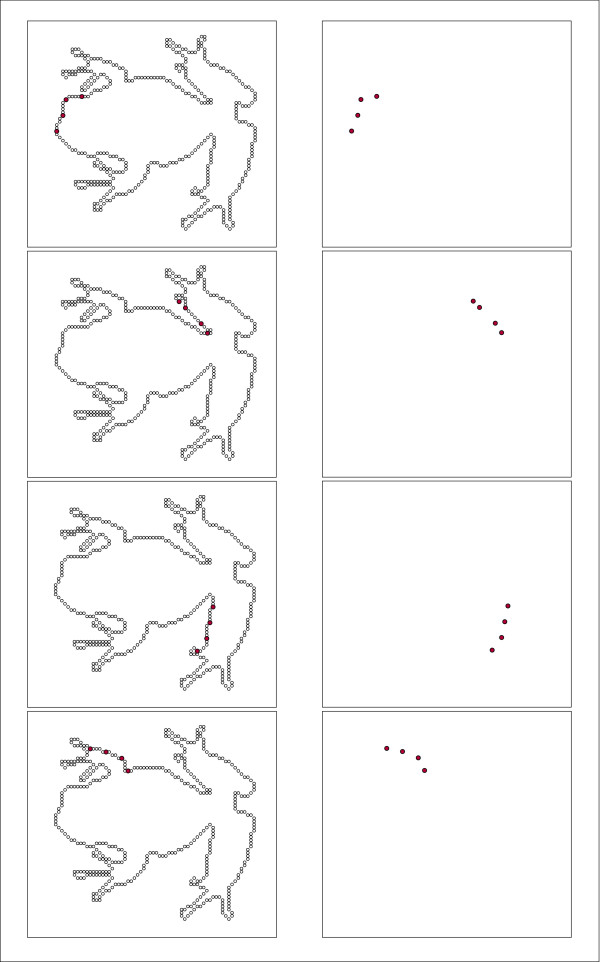
A given display set was broken at random into subsets containing four dots each (plus any residual), and these subsets were displayed successively. This figure provides examples of contiguous subsets. The left panels show the location of subset dots within the full inventory of dots. The right panels show how each subset would appear on the display board, with the time interval for display of a given subset being 0.4 ms.

The panels of Fig. 2 illustrate four subsets of contiguous dots sampled from the display set that is shown in Fig. 1. The left panels show the location of each subset, superimposed on the full complement of boundary dot positions. The right panels show how each subset would appear upon display. Each subset would be shown in rapid succession using one of the T3 intervals described below.

**Figure 3:**
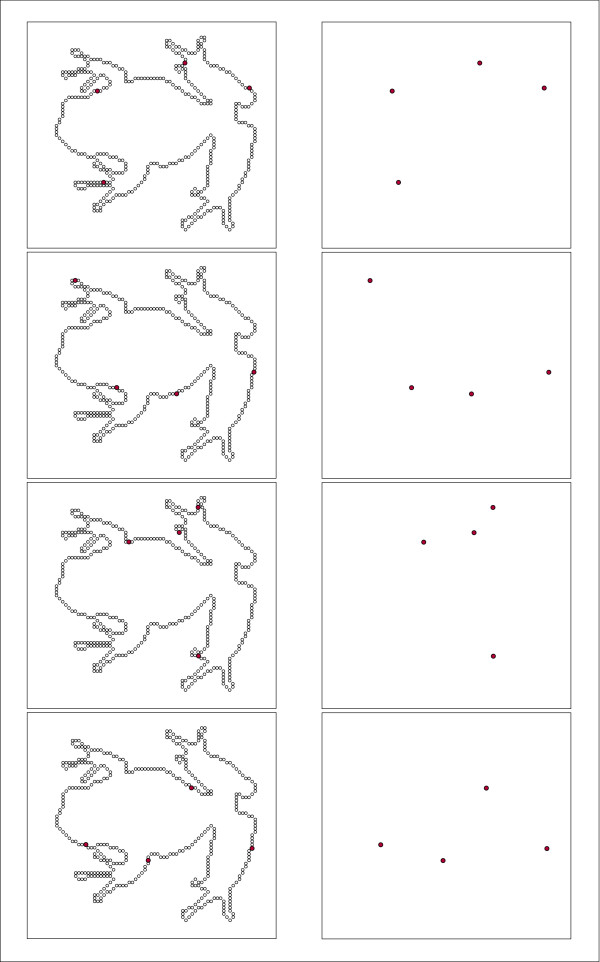
This figure provides examples of subsets in which the dots were randomly selected. Again, the left panels show the location of the subset dots within the full inventory of dots, and the right panels show how the dots in each subset would appear. Note that contour attributes, *e.g.*, orientation, curvature and length, can be seen in the contiguous subsets shown in Fig. 2, but are not present in the randomly chosen subsets shown here.

The panels of Fig. 3 provide a corresponding illustration of randomly positioned subsets that again are members of the display set shown in Fig. 1. From a comparison of the right column of panels in Figs. 2 and 3 one can see the essence of the experimental concept. The contiguous-dot subsets reflect the local contour attributes of the frog, whereas the random-dot subsets do not.

**Figure 4:**
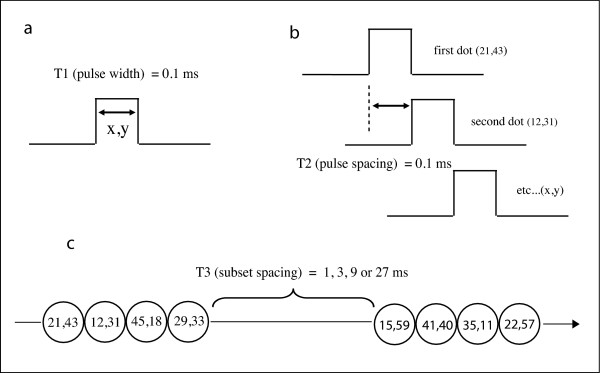
The time intervals for display of shapes are illustrated. Fig. 1a shows the duration of emission from any given LED, designated as T1, this being 0.1 ms. Fig. 1b specifies that the onset-to-onset interval of successive pulses within a given subset, designated as T2, which was also 0.1 ms. In Fig. 1c the pulses are illustrated as a string of beads. The four addresses of each subset are displayed as a group, separated by the T3 interval. For display of a given shape, theT3 interval was 1, 3, 9 or 27 ms.

Dots were displayed successively. Each dot was displayed for 0.1 ms, this being designated as T1. T2 specified the interval from onset of one address within a subset to the next address of the same subset. This was also fixed at 0.1 ms, providing for zero delay between offset of one address and onset of the next. With these T1 and T2 intervals, all addresses within a given subset were displayed in 0.4 ms. T3 specified the time interval between offset of one subset and onset of the next. There were four levels of T3, these being 1, 3, 9 and 27 ms. These time parameters are illustrated in Fig. 4.

Total display time for a given shape was determined by the number of dots in the display set multiplied by 0.1 ms, plus the T3 interval multiplied by the number of subsets minus one. For T3 = 1 ms, the minimum and maximum display times were 4.3 and 62 ms respectively, and the mean display time was 16.6 ms. For T3 = 3 ms, minimum and maximum display times (rounded up) were 10 and 150 ms, with a mean of 40 ms. For T3 = 9 ms, minimum and maximum display times were 28 and 414 ms, with a mean of 126 ms. For T3 = 27 ms, minimum and maximum display times were 82 and 1206 ms, with a mean of 320 ms.

The two levels of dot proximity and four levels of T3 provided eight treatment combinations. For each participant the inventory of 64 shapes were ranked for difficulty level, *i.e.*, the number of dots required for a 75% hit rate, and then shapes were assigned at random from the ranked list to the eight treatment combinations. The net effect of the assignment was to provide each treatment level with a sampling of shapes that were approximately equal in difficulty. Each participant saw a given shape only once, and the order for display of the shapes (and thus the treatment combinations) was random.

Eight USC undergraduates served as participants, each displaying normal or corrected to normal visual acuity. Each was naïve to the goals of the experiment, and was paid for his or her participation.

## Results

The response variable was binary, *i.e.*, recognize or failure to recognize. Participants were treated as random samples from the population of possible participants. The order of presentation of shapes was randomly specified for each participant, and the treatment combination shown for a given shape and participant was selected at random. Thus the appropriate statistical model is a Generalized Linear Mixed Model [8] with random effects of Participant and Shape, and fixed effects of Proximity and T3 interval. Logit values (log_e _(proportion/1-proportion) were calculated, and treatment differences were compared using the standard error of the difference (SED) for these values. Model predictions and standard errors of the mean for each of these predictions are shown in Table 1.

**Table 1:** Generalized Linear Mix Model Values for Treatment Conditions

	Contiguous Subsets			Random Subsets		
T3 (ms)	Mean	SEM	Backtransformed	Mean	SEM	Backtransformed

1	1.056	0.369	0.742	1.435	0.382	0.808
3	0.879	0.363	0.707	0.537	0.359	0.631
9	0.213	0.354	0.553	0.284	0.354	0.570
27	-0.442	0.358	0.391	-1.425	0.386	0.194

The Generalized Linear Mixed Model transforms the percent recognition of shapes into logit values, *i.e.*, log_e _(proportion/1-proportion). The mean logit values and the standard errors of these means are given for each T3 interval for the contiguous and random subset data. The logit values have also been backtransformed into model predictions of recognition rate.

This statistical analysis found no significant difference (p = 0.59) in recognition rate for the proximity condition, *i.e.*, recognition of shapes was not different as a function of whether the subset dots were contiguous or were at randomly selected positions.

**Figure 5:**
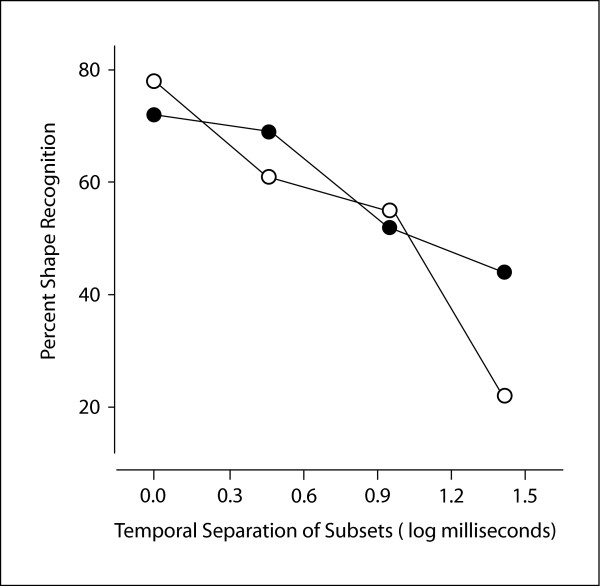
Mean percent recognition for each of the proximity conditions is plotted against the time interval separating each subset. Contiguous subsets are shown with filled circles, and random subsets are shown with open circles. The decline in recognition was significant across the tested time intervals, but the proximity conditions did not produce differential levels of shape recognition.

There was a significant (p < 0.001) linear decline in recognition rate as a function of the temporal separation between subsets, and the quadratic component was not significant (p = 0.22). The model predictions were back-transformed into values that reflect the percentage of shapes that were recognized for each of the treatment conditions. These predictions are very near the arithmetic mean recognition percentages that are plotted in Fig. 5 for contiguous and random subsets at each of the T3 intervals.

Although the difference between contiguous and random treatment conditions was not significant, inspection of the means plotted in Fig. 5 suggest the possibility that the treatments were not comparable at T3 = 27 ms. To formally evaluate this, pairwise comparisons of means were calculated, properly adjusting for the number of comparisons. There were no significant differences at the first three T3 intervals, but the difference at T3 = 27 ms was significant at p < .02. This differential could be a simple experimental artifact, in that a treatment will not always yield data that fits the overall trends.

## Discussion

A great many, perhaps a majority, of shape recognition theories propose that contour attributes, *i.e.*, orientation, curvature and linear extent, provide the elemental features that define the shape of an object. Selfridge [9] may have been the first to characterize the perceptual process in terms of an assemblage of filters, each having the ability to register a distinctive contour attribute, but many others have followed this lead [see [10-14]].

The minimal transient discrete cue (MTDC) protocol [5-7] provides a means to evaluate the validity of this hypothesis. This method briefly displays a spaced array of dots that mark the outer boundary of the shape, the number of dots being just sufficient for recognition of the shape if all of them are shown with minimal delay. By choosing which dots to sample, and introducing delays between successive samples that are chosen, one can assess the effectiveness of the shape cues being provided by the samples.

The present goal was to examine whether contiguous subsets of dots would be more effective at eliciting recognition of shapes than would subsets having an equal number of dots that were randomly chosen from the full inventory of dots. The contiguous subsets should provide a more effective stimulus for the filters that are presumed to register the contour attributes. If shapes are specified on the basis of their contour attributes, then the contiguous subsets should convey the best partial shape cues, and one would expect these subsets to be more effective for eliciting recognition.

The overall result was that contiguous and randomly selected subsets contributed equally to shape recognition, even though the randomly selected subsets did not display cues that relate to the orientation, curvature and linear extent of the boundary. This indicates that under the present test conditions, contour attributes did provide cues that are essential for shape perception.

For the present task conditions, one might speculate that information persistence allowed successive dots to accumulate, such that dots from the random subsets could eventually form contiguous strings that provided contour attributes. There is persistence of brief visual stimuli, as reported by Sperling [15], Neisser [16], Haber and Standing [17], and Eriksen and Collins [18,19], among others, and reviewed by Coltheart [20], Long [21], and Nisly and Wasserman [22]. Whereas local contour information was not provided by a given random subset, one could argue that the contour-filtering process simply waited for a number of the subsets to be delivered, after which the contour attributes could be extracted from the aggregate pool of dots.

Recent work using the present experimental protocols, however, has found that millisecond and even submillisecond differentials in the display of dot subsets can produce significant differences in shape recognition [6,7]. The result that is most critical to this discussion was provided by the second experiment in each of the cited studies, wherein the total time (and thus duration of persistence) for a given shape was held constant. Under these conditions, it was found that varying the interval between successive dots impaired recognition, with temporal separation of as little as half a millisecond being significant. Shape-relevant contour attributes are delivered directly by the contiguous dot subsets, but they could be provided by random subsets only through aggregation. The prior studies demonstrate that the cues do not aggregate without a recognition penalty.

When neural substrates for shape perception are discussed, most see the orientation-selective cells characterized by Hubel & Wiesel [3,4] as providing the first step for registering contour attributes. A given cell can be activated by a contour, and because the firing rate is influenced by the orientation, length, and (possibly) curvature of the contour, the response is thought to convey information about these attributes. It is further suggested that an assemblage of these contour filters delivers the full complement of contour attributes needed for recognition.

However, previous results from this laboratory [2] raise the question of whether shape analysis depends on activation of orientation-selective cells. That study found that recognition was possible when the full complement of dots being shown was relatively sparse. Recognition was well above chance when dot spans exceeded the length of orientation-selective receptive fields [23]. That outcome suggests that each dot is acting as an independent marker of boundary position, and that shape is defined by an unspecified – not yet known – relationship among the individual markers. Even when the orientation-selective cells are activated by an array of dots, the essential information might be the locations that have been specified rather than the collinearity in the array.

With respect to the present results, one might wonder whether the contiguous subsets were effective stimuli for the orientation-selective cells. Perhaps the cells did not respond to the very brief presentation of just four dots. There are three reasons to suggest that the subsets delivered adequate stimulation.

First, although the stimulus duration was very brief, the flashes were easily visible, *i.e.*, consciously perceived. It is generally accepted that conscious awareness of a visual stimulus requires processing by the primary visual cortex, thus the stimulus strength was adequate for activating its neurons.

Second, the span of each contiguous subset was a suitable fit to the size of receptive fields. Sceniak et al. [23] examined receptive field size of orientation-selective cells in V1 of Macaque, and found the average space constant to be 60 arc', and the average length-summation tuning curve to be 49 arc'. The four-dot array of the contiguous subsets spanned 35 arc' for horizontal or vertical alignments, and 47 arc' for diagonal alignments. Therefore each of the contiguous subsets displayed an image size that would provide four dots to the receptive fields.

Third, there is direct electrophysiological evidence that an array of briefly flashed dots will stimulate the cortical cells. Jones & Palmer [24] examined responsiveness of orientation-selective cells with successive stimulation of local points across the receptive fields, the typical duration of each stimulus being 50 ms. They reported that the responses that could be elicited by stimulating one location at a time was too weak to be of practical value in the analysis of receptive field structure. However, simultaneous activation of three sites within the receptive field yielded usable data. As indicated above, the contiguous subsets of the present experiment displayed four dots that would register on a given receptive field, and this would provide a stronger stimulus than was found to be effective by Jones & Palmer [24].

The more general point is that the random subsets as well as the contiguous subsets were seen by the subject and delivered sufficient stimulation to elicit recognition. If one took the position that the contiguous arrays provided an insufficient stimulus for activating orientation-selective cells, it would mean that recognition was accomplished without any contribution from these cells.

It is possible, that the cues used for this experiment may be especially salient for activating a primitive shape encoding system. The pattern provided by the full complement of dots is very similar to a silhouette, and recognition is best when there is maximal simultaneity of the flashed dots. This is not unlike conditions that might face an early vertebrate – perhaps a fish – who detects simultaneous movement through small openings in a wall of seaweed. The pattern that is seen could be a predator, or might be prey, and successful recognition by the creature would have implications for survival. It is likely that these recognition skills evolved, and are present in a great many present-day animals that have no cortex.

Recent evidence from this laboratory [25], gathered and published after the present research was conducted, has demonstrated that the retina contains a neural system that is sensitive to millisecond-level simultaneity when the subsets consist of dot pairs. This suggests that the present task draws on primitive shape-encoding mechanisms that put a premium on very tight temporal proximity within a stimulus pattern. More advanced image-processing systems, such as primary visual cortex, might have similar requirements for simultaneity, but with a longer time constant. This could explain the differential at T3 = 27 ms as a contribution to the temporal-integration process by orientation-selective cells that could not be accomplished in the retina.

The finding that the contour attributes did not benefit recognition under the present test conditions should not be taken as a blanket rejection of a useful role in the perception of objects. The fact that we can detect edges with a contrast differential as small as 3% speaks to the benefit of these filters for registering the presence of a boundary. Doubtless this is useful for detecting an object that is almost the same color or luminance as the background, or where it must be seen through haze. Contour filters may make it possible to see the object's boundaries under a variety of degraded conditions, and there is ample evidence that alignment of lines and edges provides a basis for object completion. It is possible, however, that this processing allows the position of discrete markers to be specified. Shape perception, *per se*, may then be based on metric relationships that have little or nothing to do with collinearity of the markers.

**Figure 6:**
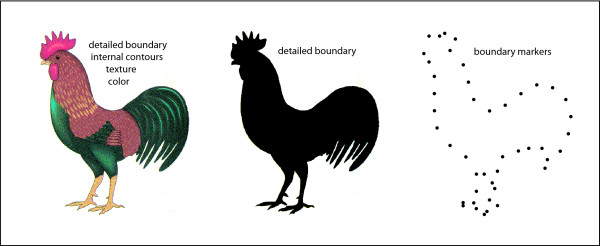
Stimuli that are available as shape cues are listed above each image. The right image provides the number of dots in a display set that allows for 75% successful recognition of the rooster when the dots are displayed successively, each being shown for 0.1 ms, and with a T3 interval of zero.

It is unclear why so many insist that shape is defined by the orientation, curvature and linear extent of the contours. We know that all manner of cues can contribute to identification of objects, but have no trouble discarding most of them as being ancillary. Fig. 6 illustrates the situation. The left image shows a detailed colored sketch that can be readily identified as a rooster. In fact the image is devoid of various depth cues that would be present in the real object. Nonetheless, we accept that the 2D image has the shape of a rooster, so the depth cues must be ancillary to our concept of shape.

The middle image has eliminated internal contours, texture, and color, replacing all these cues with uniform black. Yet this silhouette is readily identified as being in the shape of a rooster. The internal parts, color and texture must be at least somewhat ancillary, *i.e.*, nonessential.

The right image has replaced the boundary edge with an array of dots, and we can still see the stimulus as having the shape of a rooster. Contour attributes of the boundary have been eliminated, but many will insist that they must be inferred in order to identify the shape.

Previous research demonstrated that as few as 19 dots allowed for recognition of the rooster by half of the subjects [2]. It was hypothesized that the individual dots serve as markers of boundary positions, and the information needed for encoding and storage of shapes might be based on metric relationships among these markers. For the image provided by a natural object, a great many more markers are activated by its contours. But here also, some unspecified metric relationship among the markers may provide the basis for recognition, rather than collinearity of the markers.

## Conclusion

Contours provide a number of cues that might contribute to identifying a given shape. Investigators and theorists have focused on a specific set of attributes that are provided by contours, in particular suggesting that orientation, curvature and linear extent serve to characterize and specify the shape. This emphasis has been augmented by evidence that neurons in visual cortex respond more vigorously at a particular orientation of the contour, with response strength being a function of length, and in some cases, curvature. The fact that these neurons also specify location of a contour segment is given minimal attention. It is conceivable that the locations that are registered by contour filters provide the information that is most essential for characterizing a given shape.

## Abbreviations

MTDC: Minimal transient discrete cue; Arc': Minutes of visual angle; Cd/m^2^: Candela per meter squared; arc°: Degrees of visual angle; LED: Light emitting diode; Mhz: Megaherz; Log_e _: Natural log; lux: Lumen per meter squared; m: Meters; ms: Milliseconds; ns: Nanoseconds; N: Number used to specify number of dots from address list to be displayed; p: Probability; T1: Pulse width; T2: Temporal separation between members of subset pairs; T3: Temporal separation between subset pairs; V1: Primary visual cortex; 2D: Two dimensional; SEM: Standard error of the mean.

## Competing interests

The author declares that he has no competing interests.

## Authors' contributions

EG conceived of the study, designed the study, tested all participants, and wrote the article. Technical assistance for programming and data analysis was provided by contract, as noted below. EG has read and approves of the final manuscript.

## References

[B1] Kohler W (1940). Dynamics in Psychology.

[B2] Greene E (2007). Recognition of objects that are displayed with incomplete sets of discrete boundary dots. Percept Motor Skills.

[B3] Hubel DH, Wiesel TN (1959). Receptive fields of single neurons in the cat's striate cortex. J Physiol.

[B4] Hubel DH, Wiesel TN (1962). Receptive fields, binocular interactions and functional architecture in the cat's visual cortex. J Physiol.

[B5] Greene E (2006). Simultaneity in the millisecond range as a requirement for effective shape recognition. Behav Brain Funct.

[B6] Greene E (2007). Spatial and temporal proximity as factors in shape recognition. Behav Brain Funct.

[B7] Greene E (2007). Information persistence in the integration of partial cues for object recognition. Percept Psychophys.

[B8] Schall R (1991). Estimation in generalized linear models with random effects. Biometrika.

[B9] Selfridge OG, Cherry C (1957). Pattern recognition and learning. Information theory.

[B10] Sutherland NS (1968). Outlines of a theory of visual pattern recognition in animals and man. Proc R Soc Lond B Biol Sci.

[B11] Hinton GE (1981). A parallel computation that assigns canonical object-based frames of reference. Proceedings of the Seventh International Joint Conference on Artificial Intelligence.

[B12] Marr D (1982). Vision: A Computational Investigation into the Human Representation and Processing of Information.

[B13] Quinlan PT (1991). Differing approaches to two-dimensional shape recognition. Psychol Bull.

[B14] Palmer SE (1999). Vision science: photons to phenomenology.

[B15] Sperling G (1960). The information available in brief visual presentations. Psychol Monogr.

[B16] Neisser U (1967). Cognitive Psychology.

[B17] Haber RN, Standing L (1969). Direct measures of short-term visual storage. Quart J Exp Psychol.

[B18] Eriksen CW, Collins JF (1967). Some temporal characteristics of visual pattern perception. J Exp Psychol.

[B19] Eriksen CW, Collins JF (1968). Sensory traces versus the psychological moment in the temporal organization of form. J Exp Psychol.

[B20] Coltheart M (1980). Iconic memory and visible persistence. Percept Psychophys.

[B21] Long GM (1980). Iconic memory: A review and critique of the study of short-term visual storage. Psychol Bull.

[B22] Nisly SJ, Wasserman GS (1989). Intensity dependence of perceived duration: data, theories, and neural integration. Psychol Bull.

[B23] Sceniak MP, Hawken MJ, Shapley R (2001). Visual spatial characterization of macaque V1 neurons. J Neurophys.

[B24] Jones JP, Palmer LA (1987). The two-dimensional spatial structure of simple receptive fields in cat striate cortex. J Neurophys.

[B25] Greene E (2007). Retinal encoding of ultrabrief shape recognition cues. PLoS ONE.

